# Pediculosis *capitis* grave en una niña inscrita en una guardería

**DOI:** 10.7705/biomedica.4855

**Published:** 2019-12-30

**Authors:** Ángela Medina, David López, Luis Reinel Vásquez

**Affiliations:** 1 Centro de Estudios en Microbiología y Parasitología, Departamento de Medicina Interna, Facultad Ciencias de la Salud, Universidad del Cauca, Popayán, Colombia Universidad del Cauca , Departamento de Medicina Interna Facultad Ciencias de la Salud Universidad del Cauca Popayán Colombia; 2 Corporación del Laboratorio al Campo, Bogotá, D.C., Colombia Corporación del Laboratorio al Campo BogotáD.C Colombia

**Keywords:** Pediculus, infestaciones por piojos, forunculosis, anemia, niño, Colombia, Pediculus, lice infestations, furunculosis, anemia, child, Colombia

## Abstract

La *pediculosis capitis* es la ectoparasitosis más frecuente a nivel mundial. La infestación es causada por *Pediculus humanus capitis* (piojo de la cabeza) y afecta el cabello, el cuero cabelludo y la piel. Rara vez se manifiesta con otro tipo de sintomatología y, por lo general, su curso es benigno si se trata adecuadamente.

Se presenta el caso de una menor con pediculosis *capitis* de 18 meses de evolución, asociada con forúnculos, lesiones cutáneas, múltiples adenopatías y anemia, que no mejoró tras la aplicación del champú.

Inicialmente, llamó la atención la presencia de forúnculos, alopecia y adenopatías. La persistencia de la pediculosis *capitis* y el rascado intenso alteraron la integridad de la epidermis y facilitaron las infecciones secundarias por bacterias patógenas y oportunistas que produjeron impétigo, forunculosis, excoriaciones, costras hemáticas, anemia, alopecia y linfadenopatías. La pediculosis *capitis* afectó notoriamente a la paciente al causarle problemas psicológicos y de salud, agudizados por su condición económica y social. La paciente presentó manifestaciones clínicas poco frecuentes (forunculosis, anemia, fiebre, alopecia y adenopatías), lo cual se vio facilitado por la persistencia de los factores de riesgo y el hecho de que no se le inspeccionaba la cabeza ni se removían los insectos. La educación sobre los factores de riesgo y el control sanitario es indispensable para controlar la infestación.

## Abstract (en)

Pediculosis *capitis* is the most frequent ectoparasitosis around the world. The infestation is caused by *Pediculus humanus capitis* (head louse), which affects hair, scalp, and skin. It rarely presents with more symptoms and in the majority of the cases, it shows a benign course if treated properly.

We present the case of a nursery school girl with head lice infestation for 18 months. She did not improve after the shampoo treatment. This case was associated with furunculosis, skin lesions, multiple adenopathies, and anemia.

Initially, the presence of boils, alopecia, and lymphadenopathy was evident. The persistence of pediculosis *capitis* and intense scratching induced changes on skin integrity, facilitating opportunistic bacterial superinfection that led to impetiginization, furunculosis, excoriations, hematic scabs, anemia, alopecia, and lymphadenopathies. Pediculosis *capitis* affected the patient triggering psychological, economic, social, and other health problems. The patient presented uncommon symptoms (furunculosis, anemia, fever, alopecia, and adenopathies) resulting from the persistence of risk factors and the absence of head inspection and mechanical removal of insects. The education about the risk factors, as well as sanitary controls, are essential to contain the infestation.

La pediculosis es la ectoparasitosis más frecuente a nivel mundial. En los seres humanos ocasiona tres formas clínicas por la acción de tres especies de piojos: la pediculosis *corporis* (del cuerpo), la pediculosis púbica (*phthiriasis*) y la pediculosis *capitis*. Esta última es causada por el piojo de la cabeza *Pediculus humanus capitis* (familia Pediculidae), insecto hematófago del orden Phthiraptera y del suborden Anoplura. El ciclo de vida de *P. humanus capitis* se desarrolla exclusivamente en el ser humano, y afecta el cabello, el cuero cabelludo y la piel ^1^^,^^2^.

La pediculosis *capitis* ha acompañado a los humanos desde hace millones de años; probablemente, los insectos se dispersaron desde África hacia el resto del mundo por medio de las migraciones humanas ^2^. La pediculosis *capitis* se distribuye por todo el mundo, y afecta principalmente a mujeres y a niños entre los 3 y los 12 años. Cada año se presentan en el mundo 12 millones de casos nuevos de infestación por piojos de la cabeza ^3^. En Colombia no hay informes de prevalencia o incidencia de esta condición a nivel nacional o departamental, aunque se han hecho algunos estudios de prevalencia en Bogotá (8,7 %) en el 2008 ^4^ y en Popayán (11,5 %) en el 2017 ^5^. 

El prurito es el síntoma más común de la pediculosis *capitis* y se presenta por la reacción alérgica a la saliva del insecto. Sin embargo, en la mayoría de los casos esta suele ser asintomática, especialmente si no ha habido exposición previa, porque el huésped adquiere sensibilidad al antígeno del insecto cuatro a seis semanas después de entrar en contacto con su saliva ^6^^,^^7^. El prurito se manifiesta en 14 a 36 % de los casos ^6^. Los afectados rara vez presentan fiebre, malestar, irritabilidad o adenopatías cervicales u occipitales ^7^. 

El curso de la pediculosis *capitis* suele ser benigno si el manejo es oportuno, aunque el parásito puede ocasionar graves complicaciones, como infestaciones excesivas ^3^, prurito intenso, lesiones secundarias al rascado o enfermedades emergentes graves transmitidas por los mismos piojos: tifus epidémico, transmitido por *Rickettsia prowazekii*; fiebre de las trincheras y endocarditis, por *Bartonella quintana*; fiebre recurrente, por *Borrelia recurrentis*; peste, por *Yersinia pestis*, e infección por *Acinetobacter baumannii*^8^. 

El objetivo de este artículo fue presentar el caso de una menor de edad con pediculosis *capitis* de larga evolución que desencadenó graves alteraciones en su estado de salud, y resaltar la importancia de adoptar medidas de promoción, prevención y control.

## Presentación del caso

La paciente tenía cuatro años de edad y asistía al preescolar; provenía de una zona urbana de bajo nivel socioeconómico en Popayán y presentaba un cuadro clínico de 18 meses de evolución consistente en prurito intenso en cuero cabelludo, región retroauricular, orejas, cuello, nuca y cara.

Inicialmente, la niña fue tratada con champú medicado (permetrina al 1 %) aplicado por la madre durante un mes, que no logró la erradicación completa. Además, la niña refirió la presencia de numerosos piojos que se desplazaban desde la cabeza hasta su cara, espalda alta y hombros; incluso, se la observó jugando con los insectos. La niña presentaba anemia (hemoglobina: 9,5 g/dl) desde hacía seis meses, pero no había recibido tratamiento médico.

**Figura 1.**
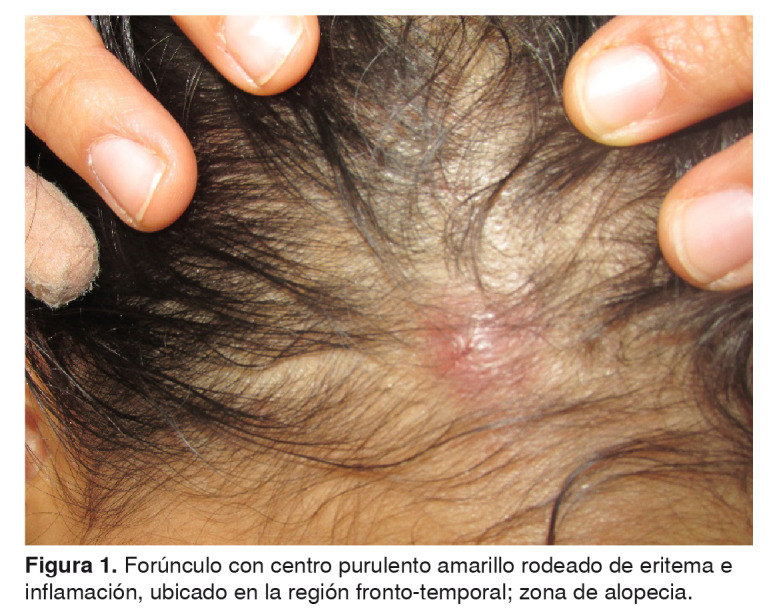
Forúnculo con centro purulento amarillo rodeado de eritema e inflamación, ubicado en la región fronto-temporal; zona de alopecia.

La madre refirió que en los cuatro meses anteriores habían aparecido lesiones en el cuero cabelludo y en la cara (figuras 1 y 2) de la niña, y había presentado picos febriles esporádicos no cuantificados, astenia y adinamia. Además, dos meses atrás, se le había diagnosticado hipotiroidismo, el cual tampoco estaba siendo tratado en el momento de la consulta.

**Figura 2.**
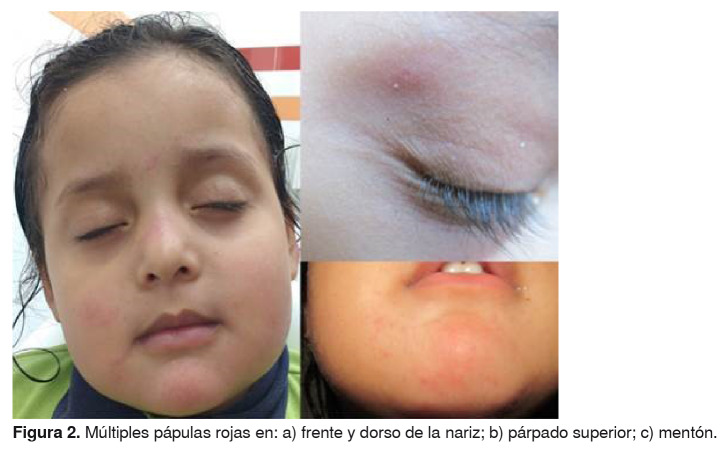
Múltiples pápulas rojas en: a) frente y dorso de la nariz; b) párpado superior; c) mentón.

**Figura 3.**
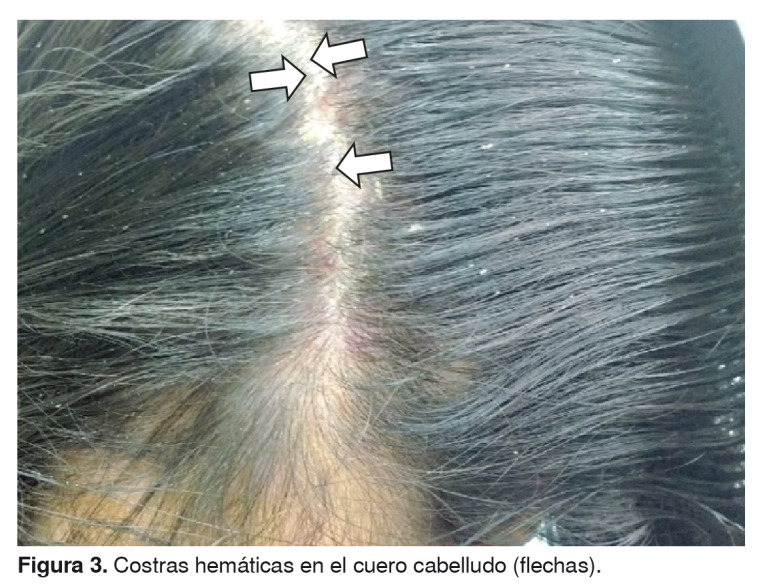
Costras hemáticas en el cuero cabelludo (flechas)

En el examen físico se encontró que la niña se mostraba irritable y presentaba palidez cutánea generalizada. En la cabeza eran visibles zonas de alopecia fronto-temporal con forúnculos de base eritematosa y centro purulento (figura 1), de olor fétido y dolorosos a la palpación, así como inflamación generalizada del cuero cabelludo, costras hemáticas (figura 3) y excoriaciones que sugerían rascado intenso. En la cara se observaron múltiples pápulas rojas, indoloras, de diferente diámetro, ubicadas en la frente, los párpados superiores, el dorso de la nariz, las mejillas y el mentón (figura 2). La niña también presentaba adenopatías retroauriculares, en la nuca, submandibulares y cervicales.

**Figura 4.**
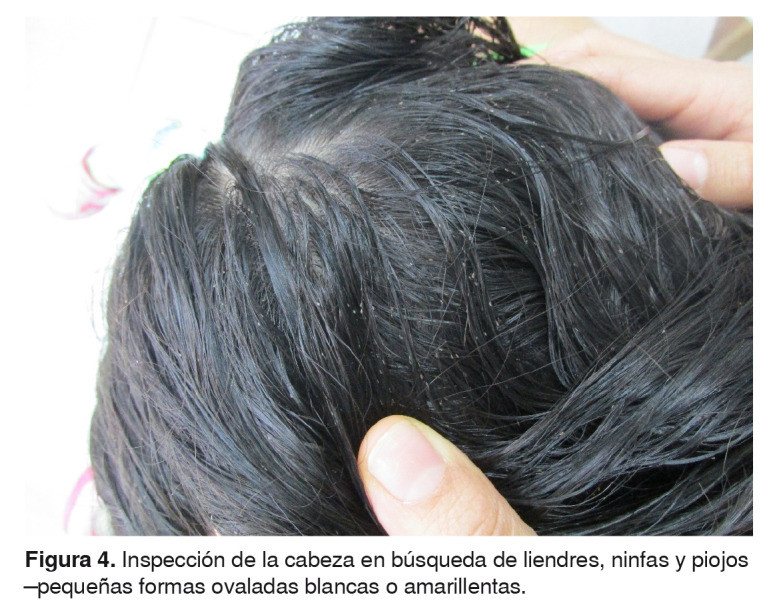
Inspección de la cabeza en búsqueda de liendres, ninfas y piojos -pequeñas formas ovaladas blancas o amarillentas.

Los insectos se extrajeron con ayuda de peines microacanalados para liendres. Se observaron abundantes liendres, ninfas y piojos adultos en las regiones frontal, parietal, temporal, occipital y retroauricular, así como en la ropa (figura 4). 

La menor y su familia viven en una casa de estrato socioeconómico 1. La vivienda tiene dos habitaciones donde duermen siete personas y la niña lo hace en una cama con tres personas más (sus padres y un hermano). La niña se baña el cabello y el cuerpo una vez por semana y comparte los peines, las toallas y los accesorios para el cabello. 

Los padres de la menor solo cursaron la primaria y ellos mismos comentaron que no inspeccionaban la cabeza de su hija, tampoco peinaban su cabello con el peine para liendres ni extraían manualmente los piojos. Además, cinco de los siete habitantes de la casa referían tener prurito en el cuero cabelludo, lo cual se confirmó por visualización de los insectos; sin embargo, la menor era la única que presentaba pediculosis *capitis* grave. 

Tres de los cinco sujetos afectados en el núcleo familiar eran adultos mayores de 25 años (dos mujeres y un hombre), y había un niño de tres años. Se les brindó educación sobre las medidas de control ambiental (no compartir peines, gorras, accesorios para el cabello, toallas ni cama) y se les explicó la necesidad de dar tratamiento a cada uno de los miembros del hogar afectados para lograr un control efectivo, pero se rehusaron a recibirlo. 

En este caso, las condiciones económicas y sociales de la familia se reflejaban en el descuido de la menor y en las limitaciones para acceder a un centro de salud donde le aplicaran el tratamiento e hicieran el seguimiento médico. En este contexto, no se cumplía con las citas de seguimiento y el acceso a la vivienda de la niña era difícil debido a problemas de orden público, por lo cual las lesiones de la piel no fueron tratadas y no se pudo verificar si la niña se curó.

## Consideraciones éticas

La madre de la menor firmó el consentimiento informado para publicar el caso; además, la niña dio su asentimiento verbal.

## Discusión

El presente caso de pediculosis *capitis* grave llama la atención, dado que rara vez se reportan casos con tal grado de compromiso dermatológico ^9^.

La pediculosis *capitis* es un problema de salud pública en Colombia y el mundo que afecta a la población sin distinción de edad, sexo o condición socioeconómica. Es la infestación más frecuente en niños, especialmente entre los 3 y los 11 años de edad ^10^^,^^11^, y tiene un efecto negativo en el individuo y en su entorno porque conlleva graves problemas psicológicos, económicos, sociales y de salud debido a las infecciones secundarias ^4^.

En este caso se hicieron visitas al hogar infantil al que acudía la niña durante tres meses. A pesar de que se eliminaron las formas móviles de *P. humanus capitis* en cada intervención, la mejoría de la niña era parcial porque los padres le aplicaban el champú antipiojos (permetrina al 1 %), pero no hacían la remoción mecánica de los insectos ni el lavado de prendas y de otros objetos capaces de transmitir los insectos. 

Según la cuidadora y la psicóloga a cargo de la guardería, la niña presentaba episodios de ansiedad, incomodidad, vergüenza, aislamiento, ausentismo escolar y efectos psicosociales. Tanto la paciente como su familia eran estigmatizadas por una comunidad convencida de que la pediculosis *capitis* se debe exclusivamente a deficiencias en los hábitos higiénicos, cuando el mayor factor de riesgo es convivir con un individuo afectado. 

El manejo de esta ectoparasitosis se dificulta en algunos casos ^4^, por lo que es necesaria la educación para su control ^3^. Se capacitó a la niña y a su familia sobre los principales factores de riesgo: tener contacto cercano con una persona infestada, el hacinamiento y el compartir peines, toallas, ropa, sombreros o accesorios para el cabello ^12^^-^^14^. Otros factores de riesgo son la ausencia o el bajo nivel de escolaridad de los cuidadores, tener bajos ingresos económicos en el hogar, bañarse la cabeza menos de tres veces a la semana y tener cabello largo ^4^^,^^5^. 

La niña de este caso pertenece a una familia de pocos ingresos económicos, lo que dificulta la compra del champú pediculicida, los peines microacanalados para liendres (indicados para desparasitar) y el transporte a los centros de salud. El cuadro clínico de la paciente puede considerarse una complicación crónica de infestación por piojos de la cabeza, agravada por el manejo inadecuado, la persistencia del riesgo y las condiciones socioeconómicas del hogar. 

A pesar de que en la literatura científica no se encuentra una definición de pediculosis *capitis* crónica o recurrente, se consideró que la niña sufría de una infestación grave, dado que esta progresó a partir del primer contacto con *P. humanus capitis*, que no hubo una nueva infestación y, tampoco, curación completa. Asimismo, hubo varios factores que favorecieron la persistencia y las complicaciones posteriores: el antecedente del uso inadecuado del champú de permetrina al 1 % (no fue formulado por un médico); el hecho de que no se hacía la extracción manual de los insectos ni se trató a los demás miembros del hogar ni el ambiente (prendas de vestir, ropa de cama); además, la niña compartía los utensilios de uso personal y se lavaba la cabeza solo una vez por semana. Asimismo, presentaba pápulas en su rostro, forúnculos y anemia, los cuales se han asociado con casos graves de pediculosis *capitis* (los piojos pueden desplazarse y descender a la cara, el cuello y el tronco superior).

El curso clínico de la paciente no se habría complicado si se hubiera hecho una intervención oportuna y adecuada. Según la literatura médica, el prurito del cuero cabelludo es la manifestación más común, pero pueden aparecer pápulas rojizas, eritematosas y pruriginosas cuatro a seis semanas después de la infestación inicial ^14^. Si la pediculosis *capitis* persiste, el rascado rara vez altera la epidermis del cuero cabelludo, pero puede causar su despigmentación y adelgazamiento, lo que propiciaría las infecciones secundarias por bacterias que llevan a desarrollar la presencia crónica de impétigo del cuero cabelludo, forunculosis, excoriaciones, costras hemáticas y linfadenopatías locales ^7^^,^^10^^,^^12^^,^^14^. La alopecia focal se ha descrito como una presentación atípica de la pediculosis *capitis* (zonas de alopecia fronto-temporal bilateral) ^6^^,^^15^. 

En cuanto a la posible asociación directa de la picadura de los piojos, o la indirecta (reacción alérgica), con las lesiones en la cara de la niña, la madre de la menor relataba que antes de que se infestara no presentaba tales lesiones, y que en algunas noches se le dificultaba conciliar el sueño, lo que atribuía a los insectos. Estas raras manifestaciones clínicas son evidentes en las fotografías de la niña.

El único antecedente patológico de la paciente era el hipotiroidismo detectado dos meses antes, el cual cursaba sin tratamiento. No se pudo determinar la causa de la anemia, pero la niña no tenía parasitismo intestinal, desnutrición ni pérdida crónica de sangre, además de la causada por la pediculosis *capitis*. Por ello, la infestación de 18 meses de evolución se podría asociar con la anemia ferropénica de la niña, asociación que se ha comprobado recientemente. Speare, *et al*., determinaron que durante una infestación grave los piojos consumen, en promedio, 20,8 ml de sangre al mes, lo que favorece el desarrollo de este tipo de anemia ^16^. Asimismo, Guss, *et al*., estudiaron durante cuatro años todos los casos graves de infestación con piojos de la cabeza y del cuerpo, y no encontraron otra explicación de la anemia, la cual se manifestaba con niveles de hemoglobina por debajo de 6 g/dl, niveles bajos de ferritina sérica e índices microcíticos de glóbulos rojos ^17^.

Se concluye que, en la pediculosis *capitis* de larga evolución asociada con un gran número de piojos, la anemia ocurre por una pérdida crónica de sangre. La infestación por piojos recurrente, prolongada o grave debe considerarse, entonces, como una posible causa de la anemia por déficit de hierro, especialmente en niños con hábitos de higiene inadecuados y condiciones socioeconómicas precarias ^7^^,^^18^, como en el caso de la presente paciente. 

### Diagnóstico

El método de elección para el diagnóstico de la pediculosis *capitis* es la inspección visual de las liendres, las ninfas o los piojos adultos. El uso del peine para liendres en el cabello húmedo facilita la remoción de los insectos, pues lentifica su desplazamiento ^1^^,^^11^. 

### Tratamiento

El tratamiento más común para la pediculosis *capitis* es la aplicación tópica de un pediculicida: la permetrina y la ivermectina son los más empleados, pues el malatión y el lindano se han asociado con reacciones adversas y neurotoxicidad ^1^^,^^19^. Sin embargo, el tratamiento más efectivo contra esta condición es la extracción manual o remoción mecánica de los insectos con un peine microacanalado para liendres, tal como lo emplearon los autores durante las visitas a la institución educativa. Este es un método fácil, seguro, económico y efectivo, aunque requiere dedicación y tiempo del cuidador ^12^. También, es importante tratar a todos los miembros del hogar, lo que en este caso no fue posible por las dificultades para desplazarse hasta la vivienda, e intervenir en el medio ambiente para evitar la transmisión de los piojos; se recomienda lavar las prendas de vestir y la ropa de cama a 50 °C, o sumergirlas en una solución pediculicida durante una hora ^14^.

### Resistencia pediculicida

Desde principios de la década de 1990 se ha evidenciado que los piojos han adquirido la capacidad de sobrevivir a productos pediculicidas (resistencia pediculicida), lo que ha contribuido al incremento de la incidencia de la infestación ^19^. Este es el caso de la mutación de los genes kdr, que codifican para los canales de sodio involucrados en los mecanismos de resistencia al efecto pediculicida de la permetrina ^20^^,^^21^.

Es probable que los insectos de la paciente hayan adquirido resistencia al principio activo del champú (permetrina), lo que explicaría la cronicidad de la infestación, aunque en este caso se ofrecieron otras opciones terapéuticas. En Colombia, no se han hecho estudios de resistencia pediculicida y, hasta el momento, no se ha determinado un protocolo de manejo de estas situaciones. Asimismo, los cuidadores manifestaron que no contaban con un adecuado servicio de salud y tampoco tenían los recursos económicos para adquirir los medicamentos. Se hizo énfasis en que los padres de la paciente siguieran practicando la remoción manual (mecánica) de los insectos. 

## Conclusiones

Este es un caso de pediculosis *capitis* grave acompañada de infecciones secundarias. La paciente presentó síntomas poco frecuentes de pediculosis *capitis* en los reportes de casos (forunculosis, anemia, fiebre, alopecia y adenopatías), resultantes de la persistencia de los factores de riesgo, y la ausencia de inspección de la cabeza y de la remoción mecánica de los insectos.

La pediculosis *capitis* es una infestación totalmente prevenible que amerita atención e intervención oportunas, debido a las múltiples complicaciones que puede ocasionar. La educación en los hogares, las escuelas y los centros de salud es fundamental para conocer los factores de riesgo, evitar la trasmisión de los piojos y concienciar a la comunidad frente a las situaciones de discriminación, estigma y diseminación de tópicos de índole psicosocial a las que se ven sometidos los afectados. En el caso de esta niña, hubo afectación psicológica por la pediculosis *capitis*.

Las entidades gubernamentales pertinentes deberían brindar una mayor atención a esta condición, así como educación sobre ella e incentivos para la investigación sobre la epidemiología y la resistencia pediculicida en cada departamento. La infestación por piojos de la cabeza debería considerarse como un problema de salud pública preocupante en Colombia.
